# Food-derived polyphenols inhibit the growth of ovarian cancer cells irrespective of their ability to induce antioxidant responses

**DOI:** 10.1016/j.heliyon.2018.e00753

**Published:** 2018-08-29

**Authors:** Youngjoo Kwon

**Affiliations:** Department of Food Science and Engineering, Ewha Womans University, Seoul, South Korea

**Keywords:** Biochemistry, Cancer research, Cell biology, Food science

## Abstract

The use of plant polyphenols to prevent cancer has been studied extensively. However, recent findings regarding the cancer-promoting effects of some antioxidants have led to reservations regarding the therapeutic use of food-derived antioxidants including polyphenols. The aim of this study was to evaluate the therapeutic potential of food-derived polyphenols and their use and safety in cancer patients. The free-radical scavenging ability of sulforaphane and various food-derived polyphenols including curcumin, epigallocatechin gallate, epicatechin, pelargonidin, and resveratrol was compared with their growth inhibitory effect on ovarian cancer cells. Oxidative stress and/or antioxidant responses and anti-proliferative pathways were evaluated after administering sulforaphane and polyphenols at doses at which they have been shown to inhibit the growth of ovarian cancer cells. No correlation was observed between their ability to scavenge free radicals and their ability to inhibit the growth of ovarian cancer cells. With the exception of epigallocatechin gallate, all of the antioxidants that were tested at doses that inhibited cell growth significantly increased NAD(P)H quinone dehydrogenase I (NQO1) expression but induced cell cycle arrest and/or apoptotic signaling. Epigallocatechin gallate exhibited a higher free radical scavenging activity but did not induce NQO1 expression at either the mRNA or at the protein level. Treatment with polyphenols at physiological doses did not significantly alter the growth of ovarian cancer cells or NQO1 expression. Therefore, individual food-derived polyphenols appear to have different anti-cancer mechanisms. Their modes of action in relation to their chemical properties should be established, rather than collectively avoiding the use of these agents as antioxidants.

## 1 Introduction

A multitude of epidemiological studies have suggested that plant foods may help reduce the risk of cancer [1]. Plant foods rich in antioxidants are thought to prevent cancer because they protect cells from damage, especially DNA damage, which can increase the risk of developing cancer [2]. Therefore, the use of food-derived antioxidants to prevent cancer has undergone extensive research.

However, the effects of antioxidants on cells appear to be more complicated than originally thought. Indeed, recent preclinical studies have suggested that some antioxidants in foods or food supplements can, in fact, stimulate the growth of existing tumors [3, 4]. Thus, oral supplementation with N-acetylcysteine (NAC) or vitamin E after the induction of lung cancer has been reported to increase tumor progression and to reduce survival in both B-RAF- and K-RAS-induced mouse models of lung cancer [3]. In addition, NAC administered after the formation of small nevi increased lymph-node metastases in a transgenic mouse model of melanoma [4]. In keeping with these preclinical results, a meta-analysis found that high consumption (20–30 mg/day) of β-carotene was significantly associated with a higher risk of lung cancer among current smokers [5]. Analogously, a large phase III randomized placebo-controlled trial concluded that dietary supplementation with vitamin E (400 IU of α-tocopheryl acetate/day) significantly increased the risk of prostate cancer in men who were otherwise healthy [6].

The observed cancer-promoting effect of these entities was again attributed to their antioxidant activity. Thus, it is thought that antioxidants may protect cancer cells from ROS-induced damage and promote their proliferation and malignancy [7]. In addition, nuclear factor erythroid 2-related factor 2 (Nrf2) and NAD(P)H:quinone oxidoreductase-1 (NQO1), a Nrf2-dependent antioxidant gene, are induced by a wide range of chemicals, including natural antioxidants [8, 9]. NQO1 and Nrf2 have been reported to be highly expressed in many types of cancer, and high expression of these genes is associated with poor prognosis [10, 11, 12]. However, the exact mechanisms underlying the reported cancer-promoting effects of antioxidants remain largely unknown. It has also been suggested that specific isoforms of vitamin E and interaction with disease conditions may be involved in the cancer-promoting effect of vitamin E [3]. Regardless of this, the cancer-promoting effect of some antioxidants has led to reservations regarding the use of antioxidant use to prevent and/or treat cancer.

The antioxidative capacities of food compounds including polyphenols are often determined by measurement of their ability to reduce 2,2-diphenyl-1-picrylhydrazyl (DPPH) and 2,2′-azino-bis(3-ethylbenzothiazoline-6-sulphonic acid) (ABTS) radicals. However, it is questionable whether food polyphenols that have been shown to have a high antioxidant capacity based on these assays exert the same antioxidant activity at the cellular or organismal level. It is also uncertain whether the anti-cancer activity of food antioxidants correlates with their antioxidant potential. Moreover, it is possible that food antioxidants exert anti-cancer effects through mechanisms that are independent of their antioxidant activity. Therefore, collectively avoiding food polyphenols as antioxidants is inappropriate, especially when the antioxidant activity of antioxidants in food is based merely on free radical scavenging assays.

Individual antioxidants may exert anti-cancer activity through different mechanisms and therefore should be considered individually rather than collectively be avoided. Determination of how the chemical properties of antioxidants including polyphenols relate to their anti-cancer mechanisms is needed, so that they can be used as single agents or in combination to increase their therapeutic efficacy [13]. In addition, improving their efficacy by combining them additively or synergistically potentially reduces the dose of antioxidants required for effective inhibition of cancer cell growth and it may overcome the problem of acquired drug resistance that is nearly ubiquitous for all current anti-cancer treatments [14]. Ovarian cancer is one of the main gynecological cancers in humans. Most ovarian cancer patients also experience disease relapse due to drug resistance, making it the most deadly gynecological malignancy [15].

This study was undertaken to evaluate the therapeutic potential of food-derived polyphenols and their use and safety in cancer patients. In this study, the radical-scavenging ability of various polyphenols derived from foods was compared with their ability to inhibit the growth of ovarian cancer cells. This allowed for examination of the relationship between the antioxidant activities of these compounds and their growth inhibitory effects. In addition, food polyphenols were evaluated for their ability to induce oxidative stress and/or antioxidant responses and to activate anti-proliferative pathways in order to test the hypothesis that antioxidants can exert an anti-cancer effect irrespective of their ability to induce antioxidant responses.

## 2 Materials and methods

### 2.1 Cell lines and cell cultures

OVCAR3, OVCAR5, and SKOV3 cells derived from human ovarian adenocarcinoma, and IHFNO-303 and IHFOT-208 fibroblasts derived from human ovaries were used. They were kindly donated by Dr. Andrew Godwin (University of Kansas Medical Center, KS, USA). Ovarian cancer cells were maintained in RPMI 1640 medium supplemented with 10% fetal bovine serum (FBS), 0.3 U/mL insulin, 2 mM L-glutamine, 100 U/mL penicillin, and 100 μg/mL streptomycin. Fibroblasts were maintained in DMEM medium supplemented with 10% FBS, 100 U/mL penicillin, and 100 μg/mL streptomycin. Cells were cultured in a humidified incubator at 37 °C and 5% CO_2_. Insulin was purchased from Life Technologies (Carlsbad, CA, USA) and all of the other reagents used for cell cultures were purchased from Thermo Fisher Scientific, Inc. (Waltham, MA, USA).

### 2.2 Antioxidants and antibodies

The antioxidants that were used comprised sulforaphane (DL-sulforaphane), curcumin (≥94% curcuminoid, ≥80% curcumin), (-)-epicatechin (≥98%), (-)-epigallocatechin gallate (≥80%), cyanidin chloride (≥80%), malvidin chloride (≥90%), pelargonidin chloride, and resveratrol (≥99%). They were all purchased from Sigma-Aldrich (St. Louis, MO, USA). All of the antioxidants were dissolved in dimethyl sulfoxide (DMSO). The final concentration of DMSO was less than 0.05%. Primary antibodies against phospho-p38, p38, phospho-ERK1/2, ERK1/2, NQO1, PARP, caspase-9, c-MYC, cyclin B1, cyclin D1, cyclin E1 and GAPDH were purchased from Cell Signaling Technology, Inc. (Danvers, MA, USA). Anti-p-JNK and anti-JNK were purchased from Santa Cruz Biotechnology, Inc. (Dallas, TX, USA).

### 2.3 Antioxidant activity

Various plant antioxidants, sulforaphane (Sul), curcumin (Cur), (-)-epicatechin (Epi), (-)-epigallocatechin-3-gallate (EGCG), cyanidin chloride (Cya), malvidin chloride (Mal), pelargonidin chloride (Pel), and resveratrol (Res) were used at concentrations ranging from 0 to 64 μM based on preliminary results to adequately compare differences in antioxidant activity across different polyphenols. DPPH (Sigma-Aldrich) dissolved in methanol was added to different concentrations of antioxidants at a final concentration of 0.1 mM [16]. The absorbance at 517 nm was measured after 15 min of incubation with DPPH.

ABTS radical cations were produced by reacting ABTS with potassium persulfate (final concentration of 7 and 2.45 mM, respectively) overnight in a dark at room temperature as described previously [17]. Once prepared, ABTS radical cations were used within three days. They were diluted with distilled water to obtain an absorbance of 1.5 at 734 nm. The final ABTS radical cation solution was added (7 times in volume of antioxidants) to different concentrations of antioxidants, and the absorbance was measured after a 15 min incubation using a VersaMax microplate reader (Molecular Device, Sunnyvale, CA, USA).

### 2.4 Cell viability assay

OVCAR3, OVCAR5, and SKOV3 cells (2,000–3,500 cells/well) in standard media containing 10% FBS were cultured overnight in flat-bottomed 96-well plates. Antioxidants at the indicated concentration or a vehicle control (0.05% DMSO) were added. After 72 h of treatment with antioxidants, cell viability was assessed using 3-[4,5-dimethylthiazol-2-yl]-2,5 diphenyl tetrazolium bromide (MTT) (Biovision Inc., Milpitas, CA, USA). After adding MTT, the plates were incubated for a further 4 h. The number of viable cells was determined by the formation of formazan as a result of the conversion of MTT by the viable cells, using a VersaMax ELISA microplate reader.

### 2.5 Thioredoxin reductase activity assay

Cells were treated with various antioxidants or vehicle for 24 h. Thioredoxin reductase activity was measured using a thioredoxin reductase activity colorimetric assay kit (Biovision Inc.) according to the manufacturer's instructions.

### 2.6 Western blot analysis

Western blot analysis was carried out as described previously [18]. Cells were lysed using radioimmunoprecipitation assay buffer (50 mM Tris HCl at pH 8.0, 5 mM EDTA, 150 mM NaCl, 1% NP40, 0.5% sodium deoxycholate, 0.1% SDS, 10 mM sodium fluoride, 1 mM sodium orthovanadate, and 1 mM β-glycerophosphate) supplemented with protease inhibitor cocktail (Sigma-Aldrich). After removing cell debris by centrifugation, the protein concentration in the resulting supernatant was measured using a Bio-Rad protein assay kit (Hercules, CA, USA). Protein samples were stored at -80 °C until analysis. Equal amounts (35 μg) of denatured proteins were separated by 10% SDS-PAGE and subjected to immunoblotting using specific primary antibodies. Protein was detected using a LumiFlash Infinity Chemiluminescence Substrate (Visual Protein Biotechnology Co., Taiwan).

### 2.7 Reverse transcription-quantitative PCR (RT-qPCR)

Total RNA was extracted from cancer cells using TRIzol reagent (Thermo Fisher Scientific, Inc.). The isolated RNA was reverse transcribed using iScript^TM^ cDNA Synthesis kit (Bio-Rad) according to the manufacturer's protocol. The primers used for the PCR were as follows: 5′-TGATATTCCAGTTCCCCCTGC-3′ and 5′- TGGCAGCGTAAGTGTAAGCA-3′ for human *NQO1*, 5′-TACAACACCCGAGCAAGGAC-3′ and 5′- GAGGCTGCTGGTTTTCCACT-3′ for human *c-MYC*, 5′-GTGAATGGACACCAACTCTACA-3′ and 5′-TAGCATGCTTCGATGTGGCA-3′ for human *Cyclin B1*, 5′-ATCAAGTGTGACCCGGACTG-3′ and 5′-CTTGGGGTCCATGTTCTGCT-3′ for human *Cyclin D1*, 5′-GCAGGATCCAGATGAAGAAATGG-3′ and 5′-TATTGTCCCAAGGCTGGCTC-3′ for human *Cyclin E1*, 5′-TCGGAGTCAACGGATTTGGT-3′ and 5′-TTCCCGTTCTCAGCCTTGAC-3′ for human *GAPDH*. Relative gene expression was determined using a CFX96 qPCR detection system (Bio-Rad) according to the manufacturer's instructions.

### 2.8 Cell cycle analysis

Cells were treated with antioxidants or vehicle for 24 h. After treatment, the cells were harvested and washed with phosphate buffered saline (PBS). The cells were fixed in ice-cold 70% ethanol for 2 h and washed with PBS. The cells were treated with RNase A (Roche, Indianapolis, USA) at a concentration of 100 μg/mL and the nuclei were stained with propidium iodide (50 μg/mL) overnight at 4 °C to measure the DNA content. The cells were suspended in 1% bovine serum albumin and subjected to cell cycle analysis using a BD FACS Calibur (BD Biosciences, San Diego, USA).

### 2.9 Cytokine analysis

Fibroblasts (400,000 cells/well) were cultured overnight in 6-well plate. Fibroblast-conditioned medium was prepared by replacing the culture supernatant with serum-free medium that contained 3.5 μM of various antioxidants or DMSO vehicle for 48 h. Culture supernatants were obtained after removal of cell debris by centrifugation at 13,000 rpm for 10 min and were used for MILLIPLEX® Multiplex assays (Millipore, Massachusetts, USA) of CXCL1, IL-6, IL-8, and MCP1. MILLIPLEX® Assays were conducted using Luminex according to the manufacturer's instructions.

### 2.10 Statistical analyses

One-way analysis of variance (ANOVA) was performed using SAS software (version 9.4, SAS institute, Inc., Cary, NC, USA) to determine the significance of the antioxidant effects. Tukey's range test was conducted to identify differences, and *p*-values less than 0.05 were considered significant.

## 3 Results and discussion

### 3.1 Different radical scavenging abilities of various food-derived polyphenols

**Fig. 1:**
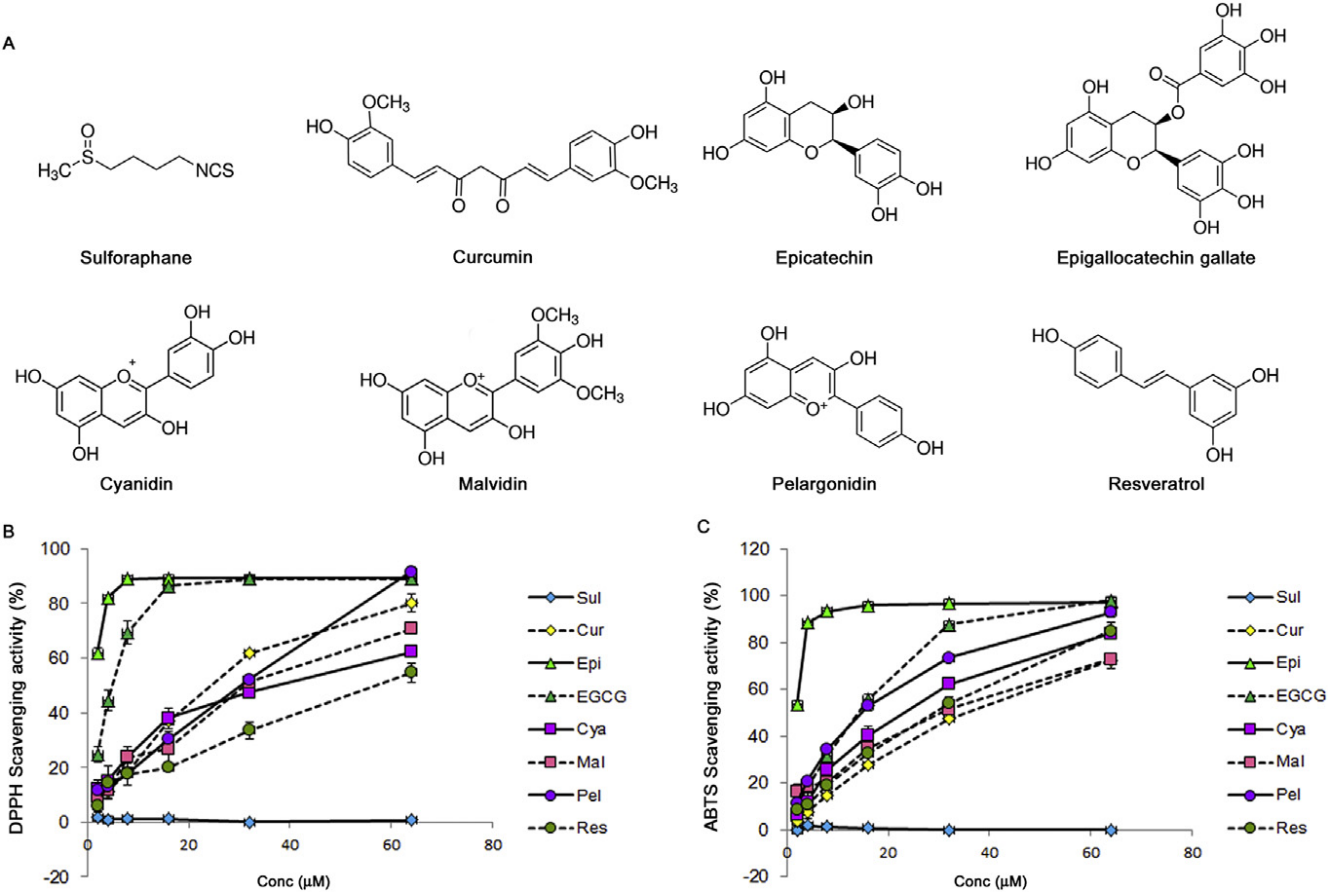
Comparison of free-radical scavenging by various food-derived polyphenols. (A) The chemical structures of sulforaphane and the various food-derived polyphenols that were tested in this study. The ability to scavenge DPPH radicals (B) and ABTS radical cation (C) was measured at final polyphenol concentrations ranging from 0 to 64 μM.

The ability of various polyphenols found in food to reduce DPPH and ABTS^+^ radicals (Fig. 1A) was measured and compared with Sul (isothiocyanate), which is known to be a strong inducer of NQO1 [19]. Sul also effectively inhibited the growth of ovarian cancer cells in our previous study [18]. Cur (curcuminoid) and Res (stilbenoid) are natural phenols. The rest of compounds are flavonoids; Epi and EGCG are classified as flavan-3-ols and Cya, Mal, and Pel are anthocyanidins.

The food-derived polyphenols exhibited various degrees of free-radical scavenging activities (Fig. 1B & C). Notably, Sul did not reduce either DPPH or ABTS^+^ as previously reported [20]. Polyphenols contained benzene rings and all of the tested polyphenols reduced both DPPH and ABTS^+^ to various degrees. The antioxidant properties of phenol-containing compounds are primarily related to the presence of aromatic rings and hydroxyl groups in the molecules and their ability to bind and neutralize free radicals [21]. Flavan-3-ols or catechin (Epi and EGCG) that have two benzene rings and a dihydropyran heterocyclic moiety with a hydroxyl group on carbon 3 had the greatest DPPH and ABTS^+^ scavenging activities (Fig. 1B & C). Tea catechins have been reported to be excellent electron donors, and their ability to scavenge radicals was similar for both DPPH and ABTS^+^ [22]. Anthocyanidins (Cya, Mal, and Pel) and phenols (Cur and Res) exhibited intermediate antioxidant activities. The DPPH- and ABTS^+^-reducing ability of these five polyphenol compounds also increased in a dose-dependent manner when used between 2 and 64 μM (Fig. 1B & C).

### 3.2 Lack of a correlation between the ability to inhibit the growth of ovarian cancer cells and the ability to scavenge free radicals by various food-derived polyphenols

**Fig. 2:**
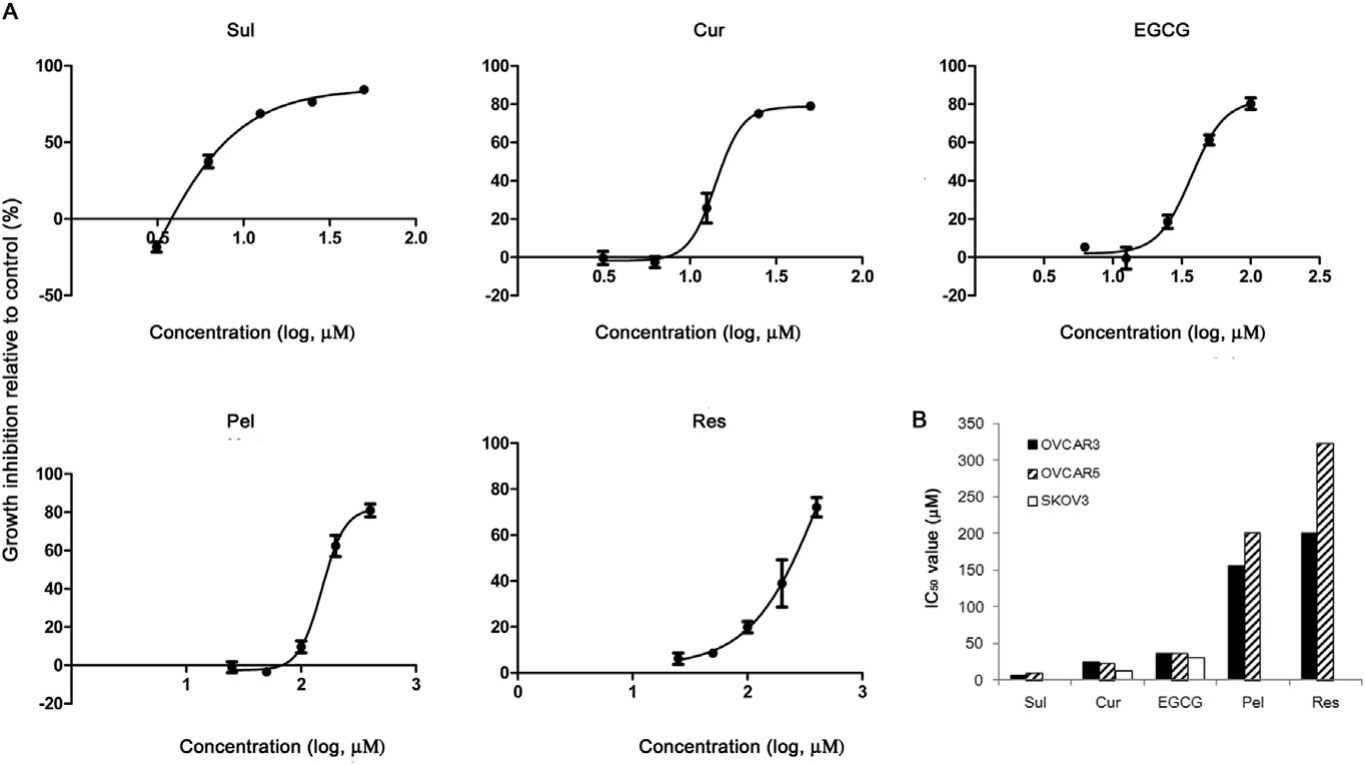
Inhibition of the growth of ovarian cancer cells by treatment with various food-derived polyphenols and sulforaphane. Ovarian cancer cells were cultured overnight in 96-well plates and then treated with 0–400 μM polyphenols and sulforaphane for 72 h. (A) Cell-growth inhibition relative to the vehicle control is shown in OVCAR3 cells. (B) The IC_50_ (half-maximal inhibitory concentration) values for OVCAR3, OVCAR5, and SKOV3 cells.

**Supplementary Figure 1:**
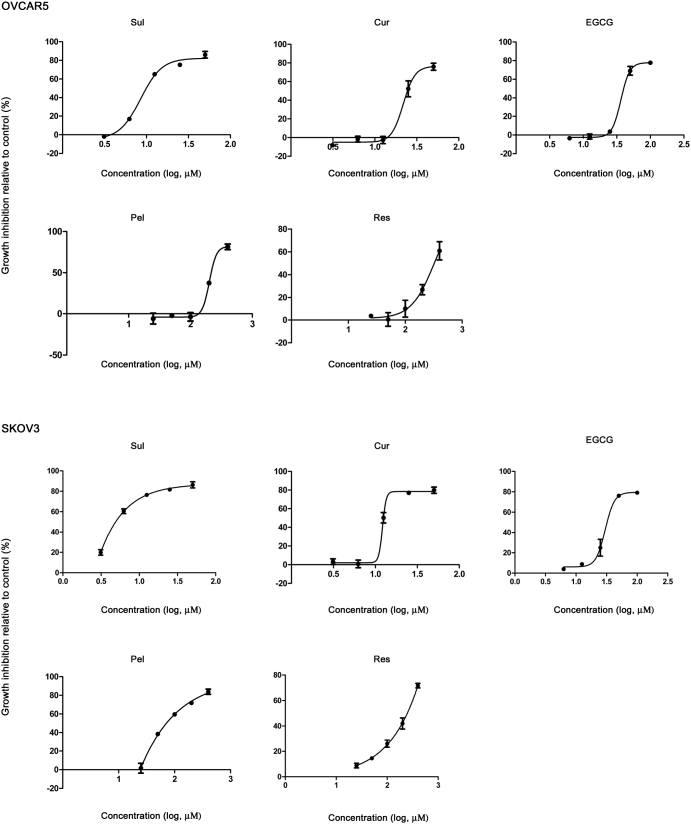
Inhibition of growth of ovarian cancer cells by treatment with various food-derived polyphenols and sulforaphane. Cell-growth inhibition relative to the vehicle control is shown in OVCAR5 and SKOV3 cells.

OVCAR3, OVCAR5, and SKOV3 cells were treated with various food antioxidants at concentrations ranging from 0 to 400 μM, and their effects on the growth of the cancer cells was evaluated. Sul, Cur, EGCG, Pel, and Res effectively reduced cell growth (the growth inhibition reached 70–80%) at the tested concentrations, although the doses required to inhibit growth of the cells differed (Fig. 2 and Supplementary Fig. 1). The half-maximal inhibitory concentration (hMED, IC_50_) was the lowest for Sul (IC_50_, 6–10 μM, Fig. 2A & B), in contrast to its inability to reduce DPPH or ABTS^+^ (Fig. 1B & C). The IC_50_ for growth-inhibition increased in the order: Cur (∼20 μM), EGCG (∼35 μM), and Pel (∼200 μM). Res had the highest IC_50_ value (>200 μM) of the antioxidants that inhibited growth of the cancer cells (Fig. 2A & B). In contrast, Epi, Cya, and Mal did not inhibit growth of the cancer cells even at concentrations as high as 400 μM (data not shown).

The chemical structures of the polyphenols alone did not predict the growth inhibitory effects of different polyphenols. Factors other than structural properties, such as size and the ability to pass through biological membranes may also contribute to their anti-cancer effects. For example, both Epi and EGCG are flavan-3-ols and both of these compounds exhibited greater free radical scavenging effects than any of the other compounds (Fig. 1). However, EGCG exhibited a growth inhibitory effect (IC_50_ values of 30–35 μM, Fig. 2) whereas Epi did not have a discernible inhibitory effect on the growth of ovarian cancer cells at concentrations less than 400 μM. Of the three anthocyanidins that were tested, only Pel inhibited the growth of ovarian cancer cells at concentrations less than 400 μM. Therefore, no correlation was observed between the extent of growth-inhibition and the radical-scavenging ability of the antioxidants among the food-derived polyphenols that were tested.

In order to assess doses that are achievable by dietary intake of these polyphenols, the effects of treatment with polyphenols on the growth of cancer cells (OVCAR3, OVCAR5, and SKOV3) were also evaluated at concentrations between 50 nM and 2 μM. At these concentrations, none of the polyphenols affected cell proliferation as assessed by MTT assay (data not shown).

### 3.3 Increased oxidative stress and/or antioxidant responses by food-derived polyphenols

Plant polyphenols exert antioxidant activity by increasing the expression or the activity of endogenous antioxidant enzymes [8]. However, natural antioxidants including polyphenols can also act as prooxidants under certain circumstances [23], and a mild oxidative stress imposed by antioxidants can induces expression of antioxidant enzymes [24].

Oxidative stress also activates mitogen-activated protein kinase (MAPK) pathways that play important roles in relaying cellular signals that lead to diverse cellular responses including cell survival, growth, and death, whereas antioxidants block MAPK activation [25]. Therefore, antioxidant treatment may trigger oxidative stress or antioxidant responses. Both the antioxidant and the pro-oxidant effects of polyphenols can modulate their anticancer potential [26]. In order to evaluate whether a dose of antioxidants that is effective at inhibiting the growth of cancer cells can induce oxidative stress or antioxidant responses, thioredoxin activity, the expression level of NQO1, and MAPK pathway activation were assessed at twice the IC_50_ (maximum effective dose, MED) and the IC_50_ (half of MED, hMED).

**Fig. 3:**
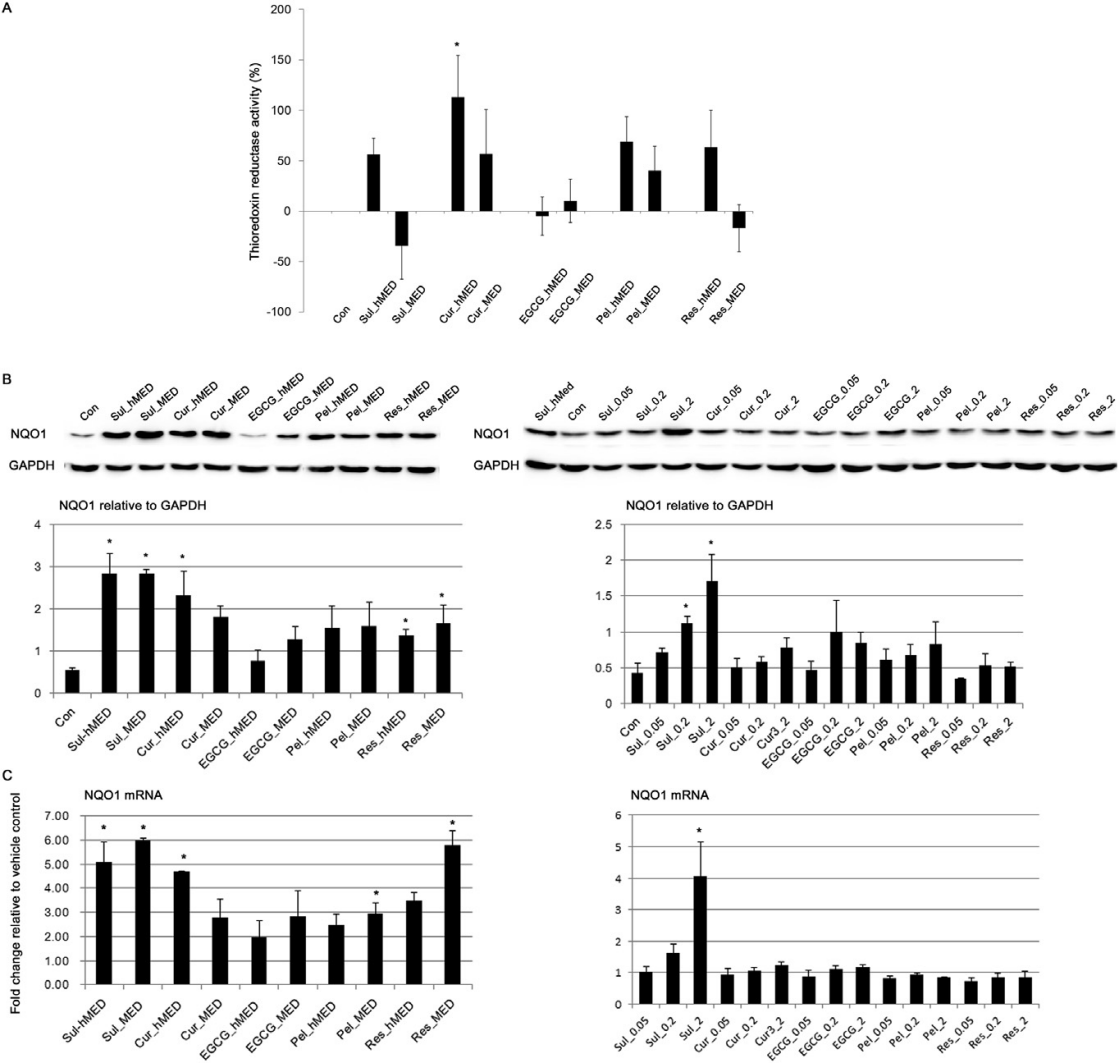
The effect of food-derived polyphenols on endogenous antioxidant enzymes. (A) Thioredoxin reductase activity was measured 24 h after treatment of OVCAR3 with food-derived polyphenols and sulforaphane at their maximal effective dose (MED) and half of MED (hMED). (B) The effect of food polyphenols and sulforaphane on the protein expression level of NQO1 was assessed by immunoblotting after treatment at the MED and hMED (left panel) and 20, 50 nM, and 2 μM of polyphenols (right panel) for 24 h. The results of the densitometric analysis of the blots are shown in the lower panel. Full, non-adjusted blots are available in Supplementary Fig. 2. (C) The NQO1 mRNA level was measured by RT-qPCR. Values are the mean ± SE, *n* = 3. An asterisk (*) represents a significant difference (*p* < 0.05) compared to the vehicle control.

Treatment with Cur for 24 h at its hMED significantly increased the thioredoxin activity of OVCAR3 cells (Fig. 3A). However, the ability of Cur to increase thioredoxin activity was reduced by half at the MED compared to its hMED, which is not significantly different from the vehicle control (Con). The high induction of thioredoxin activity at the hMED but not at the MED may warrant further study to determine whether it may be related to the resistance of cancer cells to Cur treatment [27].

Treatment with antioxidants increased the protein expression level of NQO1 (Fig. 3B). As reported previously, the increase in the NQO1 expression level was greater with Sul treatment [12, 19, 28]. Treatment with Cur and Res also greatly induced NQO1 expression (Fig. 3B). Interestingly, EGCG treatment did not significantly increase NQO1 expression (Fig. 3B) although EGCG exhibited a high free radical scavenging activity (Fig. 1). The induction of NQO1 expression by polyphenols was also examined at physiologically achievable doses. At physiologically achievable doses, the polyphenols that were tested did not significantly change the level of NQO1 expression (Fig. 3B). However, treatment with Sul (50 nM and 2 μM) significantly increased the level of NQO1 expression; treatment with 2 μM Sul increased NQO1 expression as did the hMED (6.25 μM) (Fig. 3B). Overall, a similar tendency was also observed in terms of mRNA levels at both therapeutic and physiological concentrations (Fig. 3C). Therefore, with the exception of EGCG, the polyphenols that were tested induced NQO1 expression at therapeutically effective doses. Whether the efficacy of anti-cancer drugs can be altered by high dietary consumption of foods rich in Sul requires further study.

**Fig. 4:**
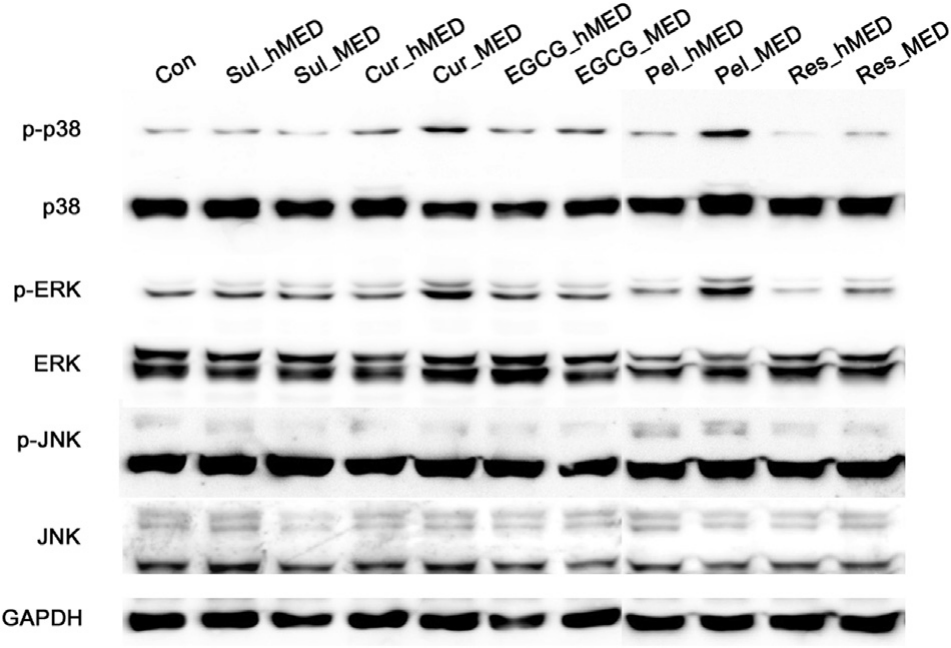
Activation of mitogen-activated protein kinases (MAPKs) by food-derived polyphenols. Activation of MAPKs [p38; extracellular signal-regulated kinase (ERK); c-Jun NH2-terminal kinase (JNK)] in OVCAR3 cells was assessed by immunoblotting after a 6 h treatment at the maximal effective dose (MED) and half of MED (hMED) of the polyphenols and sulforaphane. The data are representative of three independent experiments. GAPDH, glyceraldehyde-3-phosphate dehydrogenase. Full, non-adjusted blots are available in Supplementary Fig. 2.

Phosphorylation of both p38 and extracellular signal-regulated kinase (ERK) was induced when Cur and Pel were used. This increase in the phosphorylation of both p38 and ERK was dose-dependent (Fig. 4). All of the antioxidants induced phosphorylation of ERK2 (44 kD) in particular, although the extent of this induction was limited. ERK2 activation has previously been shown to be related to intracellular oxidation [29]. Phosphorylation of c-Jun NH_2_-terminal kinase (JNK) was not noticeably induced by treatment with any of the antioxidants (Fig. 4). Hence, some polyphenols may also induce oxidative stress. It is also possible that endogenous antioxidant enzymes (e.g., NQO1) are induced in response to increased intracellular ROS levels or alteration of the cellular redox state [24, 26]. Sul-induced inhibition of the growth of cancer cells has been shown to be related to Sul-induced oxidation that can be prevented by a thiol-reducing agent [18].

### 3.4 Activation of anti-proliferative pathways by food-derived polyphenols

**Fig. 5:**
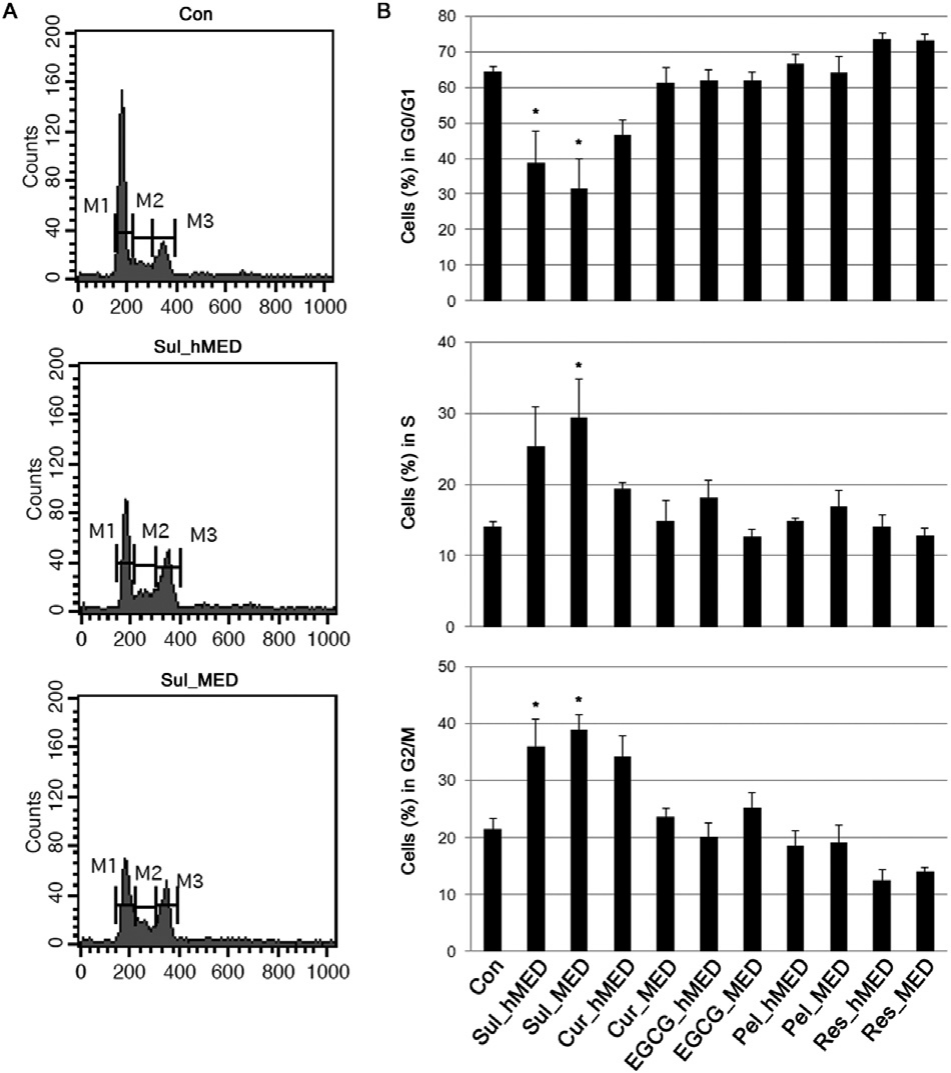
The effect of food-derived polyphenols on cell cycle progression. OVCAR3 cells were treated with polyphenols and sulforaphane at their maximal effective doses (MED) and half of MED (hMED) and the DNA content of the cells was analyzed by flow cytometry. (A) Representative flow cytometry plots. (B) The percentage of cells in each phase of the cell cycle. Values are the mean ± SE, *n* = 3. An asterisk (*) represents a significant difference (*p* < 0.05) compared to the vehicle control. M1, G0/G1 phase; M2, S phase; M3, G2/M phase.

The growth of cancerous cells can be inhibited by blocking cell cycle progression. The effect of various antioxidants on cell cycle progression was examined. Cells were treated with various antioxidants at their hMEDs or MEDs for 24 h, and the fraction of the cell population in each phase of the cell cycle was assessed by measurement of their DNA content. OVCAR3 cells treated with Sul at both the hMED and the MED accumulated in G2/M phase with a corresponding reduction in the number of cells in G0/G1 phase (Fig. 5). Cur treatment at the hMED led to slight increase in the number of cells in G2/M phase. However, this effect of Cur was not significantly different from that of Con, and it was reduced by treatment at the MED (Fig. 5). Therefore, of the antioxidants that were tested, only Sul treatment resulted in a significant degree of G2/M phase arrest.

**Fig. 6:**
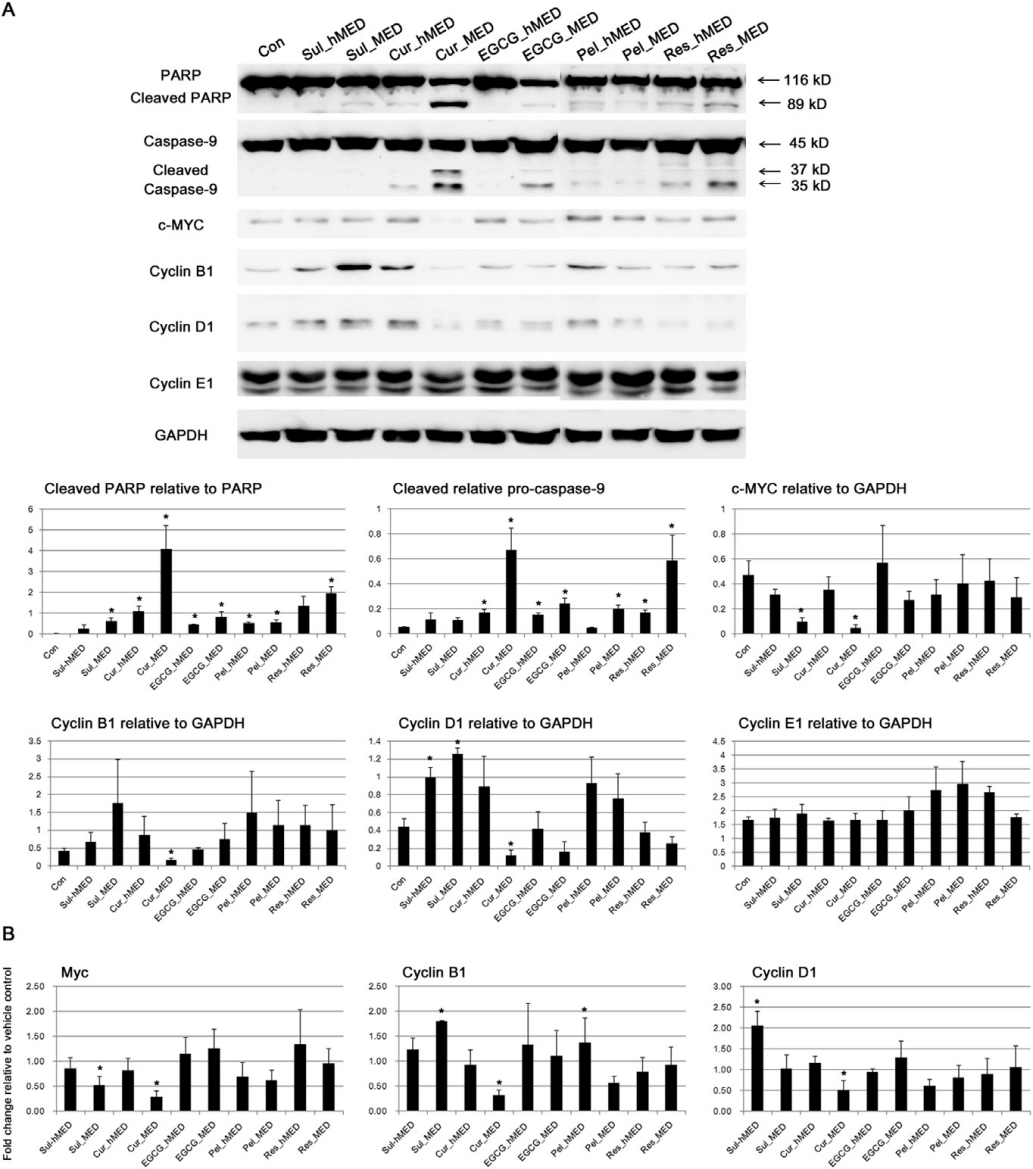
Induction of anti-proliferative pathways by food-derived polyphenols. (A) The effect of food polyphenols on protein expression levels was assessed by immunoblotting after 24 h of treatment at the MED and hMED of the polyphenols and sulforaphane. The results for the densitometric analysis of blots are shown in the lower panel. PARP, poly (ADP-ribose) polymerase (PARP); GAPDH, glyceraldehyde-3-phosphate dehydrogenase. Full, non-adjusted blots are available in Supplementary Fig. 2. (B) Transcription levels of the indicated genes were measured by RT-qPCR. Values are the mean ± SE, *n* = 3. An asterisk (*) represents a significant difference (*p* < 0.05) compared to the vehicle control.

Next, it was examined whether treatment with antioxidants altered the expression level of proteins involved in cell proliferation and apoptosis. Treatment with all of the antioxidants that were tested increased the proteolytic cleavage of poly (ADP-ribose) polymerase (PARP) (Fig. 6A). With the exception of Sul, treatment with the antioxidants also induced cleavage of caspase-9 (Fig. 6A). Sul induced less apoptosis than the polyphenols in the current study, in contrast to the strong induction of apoptosis in number of other studies [30, 31, 32, 33]. The ability of Sul to induce apoptosis was dose-dependent [30, 31]. In these studies that demonstrated induction of apoptosis by Sul, relatively high concentrations of Sul were used such as 20 μM in PC-3 cells [32] and 30 μM in Jurkat T leukemia cells [33]. In addition, cell death induced by Sul appeared to be mainly by necrosis with only a small amount of apoptosis [34]. Therefore, treatment with antioxidants at the hMED and the MED induced anti-proliferative pathways in accordance with their growth inhibitory effects (Fig. 2A & B).

Of note, treatment with either Sul or Cur, which greatly induced NQO1 expression (Fig. 3B & C), also suppressed expression of c-MYC at both the protein and the mRNA level (Fig. 6A & B). However, their effects on Cyclin B1 and Cyclin D1 were different; Cur treatment reduced the expression level of Cyclin B1 and Cyclin D1 whereas treatment with Sul increased their expression (Fig. 6A & B). Previous studies also demonstrated that Sul could induce G2/M arrest by inactivation of the Cyclin B1 and Cdc2 complex without altering expression level of Cyclin B1 in ovarian cancer cells [35] and with increased protein expression of Cyclin B1 in acute lymphoblastic leukemia cells [36]. Sul increased Cyclin D1 protein expression when it induced G2/M arrest, and Cyclin D1 overexpression has been reported to be required for Sul-induced apoptotic and necrotic cell death in non-small lung cancer cells [34]. Although exact mechanisms involved still need to be elucidated, this study clearly demonstrated that the anti-cancer mechanisms of Cur are quite different from those of Sul. At physiological doses (20, 50 nM, and 2 μM), the polyphenols and Sul did not alter the protein or the mRNA levels of any of the genes (*c-Myc*, *Cyclin B1*, and *Cyclin D1*) that were tested in this study (data not shown).

### 3.5 Modulation of the secretion of growth factors derived from fibroblasts by polyphenols

The growth of cancer cells is largely influenced by fibroblasts that consist of tumor microenvironment [37]. Fibroblasts stimulate the growth and malignancy of cancer cells by providing a variety of growth factors and cytokines, including CXCL1, IL-6, IL-8, and MCP1 [38, 39, 40, 41]. Changes in the secretion of these cytokines by fibroblasts were evaluated by assaying their levels following treatment with polyphenols. The polyphenols were used at a concentration of 3.5 μM to avoid inhibition of the growth of fibroblasts under the serum-free conditions.

**Fig. 7:**
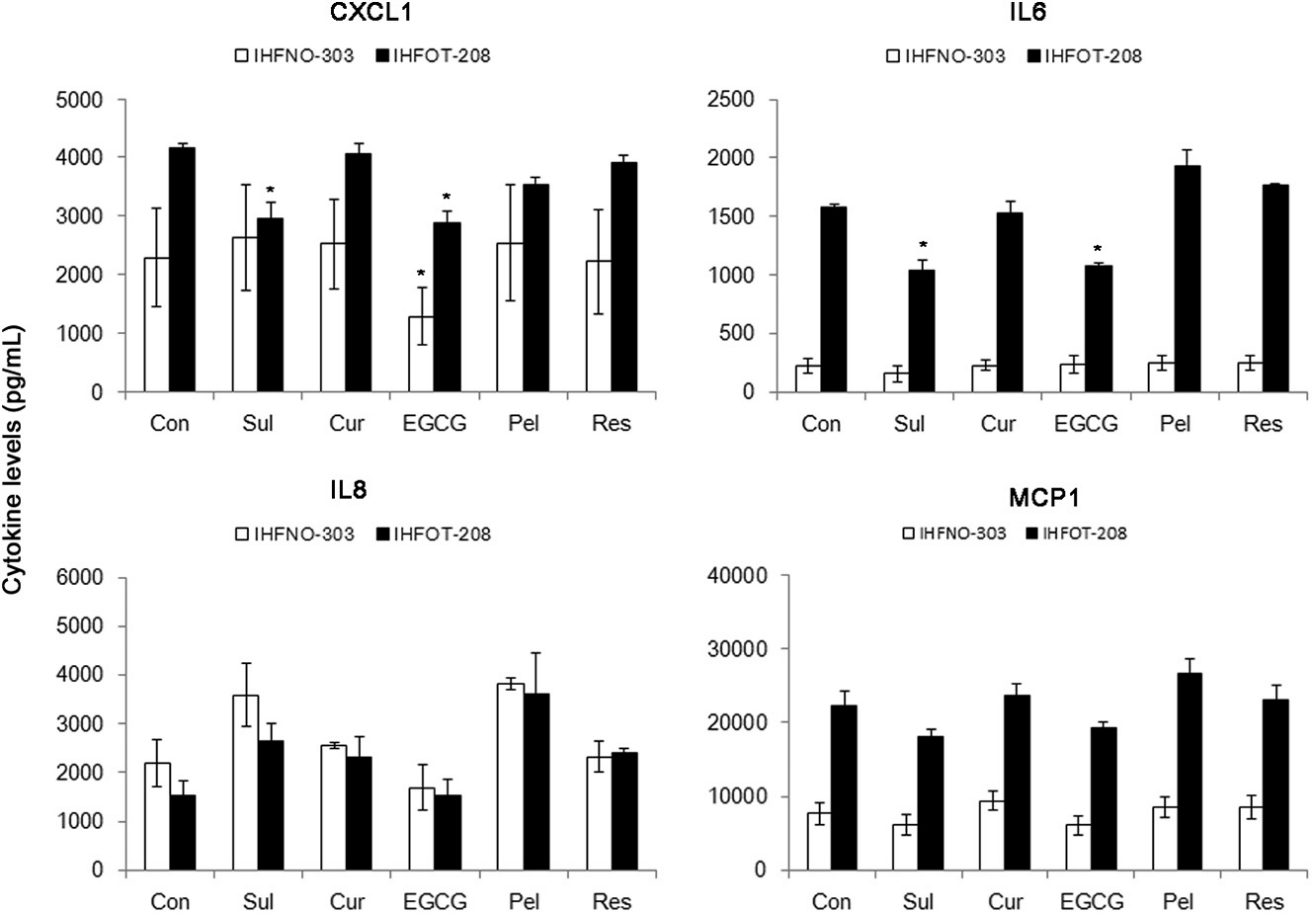
The effect of food-derived polyphenols on the levels of cytokines secreted by fibroblasts. Serum-starved fibroblasts were treated with polyphenols and sulforaphane (3.5 μM), and changes in the levels of cytokines secreted by the fibroblasts were measured. Values are the mean ± SE, *n* = 3. An asterisk (*) represents a significant difference (*p* < 0.05) compared to the vehicle control.

IHFOT-208 fibroblasts secreted higher levels of the indicated cytokines compared to IHFNO-303 fibroblasts (Fig. 7). Treatment with EGCG significantly reduced the secretion of CXCL1 by IHFNO-303 and IHFOT-208 fibroblasts (Fig. 7). Sul also inhibited the secretion of CXCL1, but only by IHFOT-208, without significantly altering its secretion by IHFNO-303 fibroblasts (Fig. 7). Treatment with both Sul and EGCG also significantly reduced the level of IL-6 secreted by IHFOT-208 fibroblasts, which secreted much more IL-6 than IHFNO-303 fibroblasts (Fig. 7). The levels of secreted IL-8 and MCP1 were not significantly altered by treatment with any of the antioxidants. Therefore, it is possible that food-derived polyphenols can suppress the growth of cancer cells by inhibition of the secretion or the production of cytokines derived from the tumor microenvironment.

## 4 Conclusions

The ability to scavenge free radicals has been widely used to determine the antioxidant activity of plant extracts and polyphenols. However, the ability to scavenge free radicals did not correlate with the ability of polyphenols to inhibit the growth of ovarian cancer cells or the ability to induce NQO1 expression. Food-derived polyphenols at their hMED and MED inhibited the growth of ovarian cancer cells by the induction of anti-proliferative pathways, irrespective of whether they induced oxidative stress and/or antioxidant responses. Furthermore, some antioxidants (e.g., EGCG and Sul) reduced the amount of cytokines secreted by fibroblasts, thus indicating that they can suppress cytokine-induced growth promotion *in vivo*. Therefore, the growth inhibitory effect of polyphenols does not necessarily require the absence of antioxidant responses. Individual polyphenols should be considered to have different anti-cancer mechanisms rather than being collectively avoided as antioxidants.

In terms of the physiologically achievable dose, most antioxidants may be able to yield beneficial effects when used in combination with other food antioxidants that can act synergistically or through additive mechanisms on their efficacy. Therefore, it will ultimately be important to establish the relationship between the chemical nature and the anti-cancer mechanisms of various plant antioxidants in order to adequately combine them in ways that are beneficial to cancer patients.

## Declarations

### Author contribution statement

Youngjoo Kwon: Conceived and designed the experiments; Performed the experiments; Analyzed and interpreted the data; Wrote the paper.

### Funding statement

This work was supported by a grant from the National Research Foundation of Korea (NRF), funded by the Ministry of Science, ICT & Future Planning (2014R1A1A3050916).

### Competing interest statement

The author declares no conflict of interest.

### Additional information

No additional information is available for this paper.

## Appendix A Supplementary data

Supplementary Figure 2 _spl_Full length blots for Figure 346_spl_ Supplementary Figure 2 _spl_Full length blots for Figure 346_spl_

## References

[1] Key T.J., Schatzkin A., Willett W.C., Allen N.E., Spencer E.A., Travis R.C. (2004). Diet, nutrition and the prevention of cancer. Publ. Health Nutr..

[2] Collins A.R. (2005). Antioxidant intervention as a route to cancer prevention. Eur. J. Cancer.

[3] Sayin V.I., Ibrahim M.X., Larsson E., Nilsson J.A., Lindahl P., Bergo M.O. (2014). Antioxidants accelerate lung cancer progression in mice. Sci. Transl. Med..

[4] Le Gal K., Ibrahim M.X., Wiel C., Sayin V.I., Akula M.K., Karlsson C. (2015). Antioxidants can increase melanoma metastasis in mice. Sci. Transl. Med..

[5] Tanvetyanon T., Bepler G. (2008). Beta-carotene in multivitamins and the possible risk of lung cancer among smokers versus former smokers: a meta-analysis and evaluation of national brands. Cancer.

[6] Klein E.A., Thompson I.M., Tangen C.M., Crowley J.J., Lucia M.S., Goodman P.J. (2011). Vitamin E and the risk of prostate cancer: the selenium and vitamin E cancer prevention trial (SELECT). J. Am. Med. Assoc..

[7] Hawk M.A., McCallister C., Schafer Z.T. (2016). Antioxidant activity during tumor progression: a necessity for the survival of cancer cells?. Cancers (Basel).

[8] Anantachoke N., Lomarat P., Praserttirachai W., Khammanit R., Mangmool S. (2016). Thai fruits exhibit antioxidant activity and induction of antioxidant enzymes in HEK-293 cells. Evid. Based Compl. Alternat. Med..

[9] Stefanson A.L., Bakovic M. (2014). Dietary regulation of Keap1/Nrf2/ARE pathway: focus on plant-derived compounds and trace minerals. Nutrients.

[10] Lin L., Sun J., Tan Y., Li Z., Kong F., Shen Y. (2017). Prognostic implication of NQO1 overexpression in hepatocellular carcinoma. Hum. Pathol..

[11] Ma Y., Kong J., Yan G., Ren X., Jin D., Jin T. (2014). NQO1 overexpression is associated with poor prognosis in squamous cell carcinoma of the uterine cervix. BMC Cancer.

[12] Sporn M.B., Liby K.T. (2012). NRF2 and cancer: the good, the bad and the importance of context. Nat. Rev. Cancer.

[13] Kwon Y. (2014). Curcumin as a cancer chemotherapy sensitizing agent. J. Korean Soc. Appl. Biol. Chem..

[14] Al-Lazikani B., Banerji U., Workman P. (2012). Combinatorial drug therapy for cancer in the post-genomic era. Nat. Biotechnol..

[15] Torre L.A., Bray F., Siegel R.L., Ferlay J., Lortet-Tieulent J., Jemal A. (2015). Global cancer statistics, 2012. CA Cancer J. Clin..

[16] Olugbami J.O., Gbadegesin M.A., Odunola O.A. (2015). In vitro free radical scavenging and antioxidant properties of ethanol extract of Terminalia glaucescens. Pharmacogn. Res..

[17] Ahmed F.A., Ali R.F. (2013). Bioactive compounds and antioxidant activity of fresh and processed white cauliflower. BioMed Res. Int..

[18] Kim S.C., Choi B., Kwon Y. (2017). Thiol-reducing agents prevent sulforaphane-induced growth inhibition in ovarian cancer cells. Food Nutr. Res..

[19] Ghorab M.M., Alsaid M.S., Higgins M., Dinkova-Kostova A.T., Shahat A.A., Elghazawy N.H. (2016). NAD(P)H:quinone oxidoreductase 1 inducer activity of some novel anilinoquinazoline derivatives. Drug Des. Dev. Ther..

[20] Farag M.A., Abdel Motaal A.A. (2010). Sulforaphane composition, cytotoxic and antioxidant activity of crucifer vegetables. J. Adv. Res..

[21] Bors W., Michel C., Stettmaier K. (2001). Structure-activity relationships governing antioxidant capacities of plant polyphenols. Methods Enzymol..

[22] Lee L.S., Kim S.H., Kim Y.B., Kim Y.C. (2014). Quantitative analysis of major constituents in green tea with different plucking periods and their antioxidant activity. Molecules.

[23] Eghbaliferiz S., Iranshahi M. (2016). Prooxidant activity of polyphenols, flavonoids, anthocyanins and carotenoids: updated review of mechanisms and catalyzing metals. Phytother Res..

[24] Prochazkova D., Bousova I., Wilhelmova N. (2011). Antioxidant and prooxidant properties of flavonoids. Fitoterapia.

[25] Son Y., Cheong Y.K., Kim N.H., Chung H.T., Kang D.G., Pae H.O. (2011). Mitogen-activated protein kinases and reactive oxygen species: how can ROS activate MAPK pathways?. J. Signal Transduct..

[26] Catanzaro E., Calcabrini C., Turrini E., Sestili P., Fimognari C. (2017). Nrf2: a potential therapeutic target for naturally occurring anticancer drugs?. Expert Opin. Ther. Targets.

[27] Bhatia M., McGrath K.L., Di Trapani G., Charoentong P., Shah F., King M.M. (2016). The thioredoxin system in breast cancer cell invasion and migration. Redox Biol..

[28] Zhang C., Su Z.Y., Khor T.O., Shu L., Kong A.N. (2013). Sulforaphane enhances Nrf2 expression in prostate cancer TRAMP C1 cells through epigenetic regulation. Biochem. Pharmacol..

[29] Goldstone S.D., Hunt N.H. (1997). Redox regulation of the mitogen-activated protein kinase pathway during lymphocyte activation. Biochim. Biophys. Acta.

[30] Chuang L.T., Moqattash S.T., Gretz H.F., Nezhat F., Rahaman J., Chiao J.W. (2007). Sulforaphane induces growth arrest and apoptosis in human ovarian cancer cells. Acta Obstet. Gynecol. Scand..

[31] Geng Y., Zhou Y., Wu S., Hu Y., Lin K., Wang Y. (2017). Sulforaphane induced apoptosis via promotion of mitochondrial fusion and ERK1/2-mediated 26S proteasome degradation of novel pro-survival bim and upregulation of bax in human non-small cell lung cancer cells. J. Cancer.

[32] Singh A.V., Xiao D., Lew K.L., Dhir R., Singh S.V. (2004). Sulforaphane induces caspase-mediated apoptosis in cultured PC-3 human prostate cancer cells and retards growth of PC-3 xenografts in vivo. Carcinogenesis.

[33] Fimognari C., Lenzi M., Sciuscio D., Cantelli-Forti G., Hrelia P. (2007). Cell-cycle specificity of sulforaphane-mediated apoptosis in Jurkat T-leukemia cells. In Vivo.

[34] Zuryn A., Litwiniec A., Safiejko-Mroczka B., Klimaszewska-Wisniewska A., Gagat M., Krajewski A. (2016). The effect of sulforaphane on the cell cycle, apoptosis and expression of cyclin D1 and p21 in the A549 non-small cell lung cancer cell line. Int. J. Oncol..

[35] Chang C.C., Hung C.M., Yang Y.R., Lee M.J., Hsu Y.C. (2013). Sulforaphane induced cell cycle arrest in the G2/M phase via the blockade of cyclin B1/CDC2 in human ovarian cancer cells. J. Ovarian Res..

[36] Suppipat K., Park C.S., Shen Y., Zhu X., Lacorazza H.D. (2012). Sulforaphane induces cell cycle arrest and apoptosis in acute lymphoblastic leukemia cells. PLoS One.

[37] Kwon Y., Godwin A.K. (2017). Regulation of HGF and c-MET interaction in normal ovary and ovarian cancer. Reprod. Sci..

[38] Bolitho C., Hahn M.A., Baxter R.C., Marsh D.J. (2010). The chemokine CXCL1 induces proliferation in epithelial ovarian cancer cells by transactivation of the epidermal growth factor receptor. Endocr. Relat. Cancer.

[39] Lederle W., Depner S., Schnur S., Obermueller E., Catone N., Just A. (2011). IL-6 promotes malignant growth of skin SCCs by regulating a network of autocrine and paracrine cytokines. Int. J. Cancer.

[40] Chavey C., Muhlbauer M., Bossard C., Freund A., Durand S., Jorgensen C. (2008). Interleukin-8 expression is regulated by histone deacetylases through the nuclear factor-kappaB pathway in breast cancer. Mol. Pharmacol..

[41] Furukawa S., Soeda S., Kiko Y., Suzuki O., Hashimoto Y., Watanabe T. (2013). MCP-1 promotes invasion and adhesion of human ovarian cancer cells. Anticancer Res..

