# Supplementary figures and images for: Food-derived polyphenols inhibit the growth of ovarian cancer cells irrespective of their ability to induce antioxidant responses
Source: Heliyon. 2018 Aug 29;4(8):e00753. doi: 10.1016/j.heliyon.2018.e00753 (PMC6121158; doi:10.1016/j.heliyon.2018.e00753)

Figure 3

Left panel

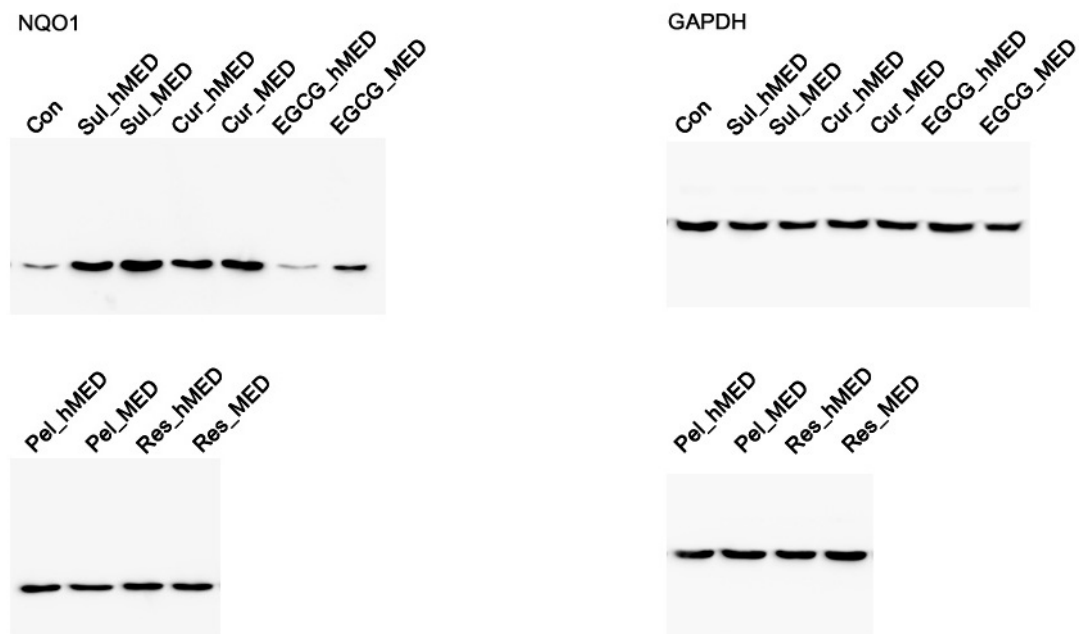

Right panel

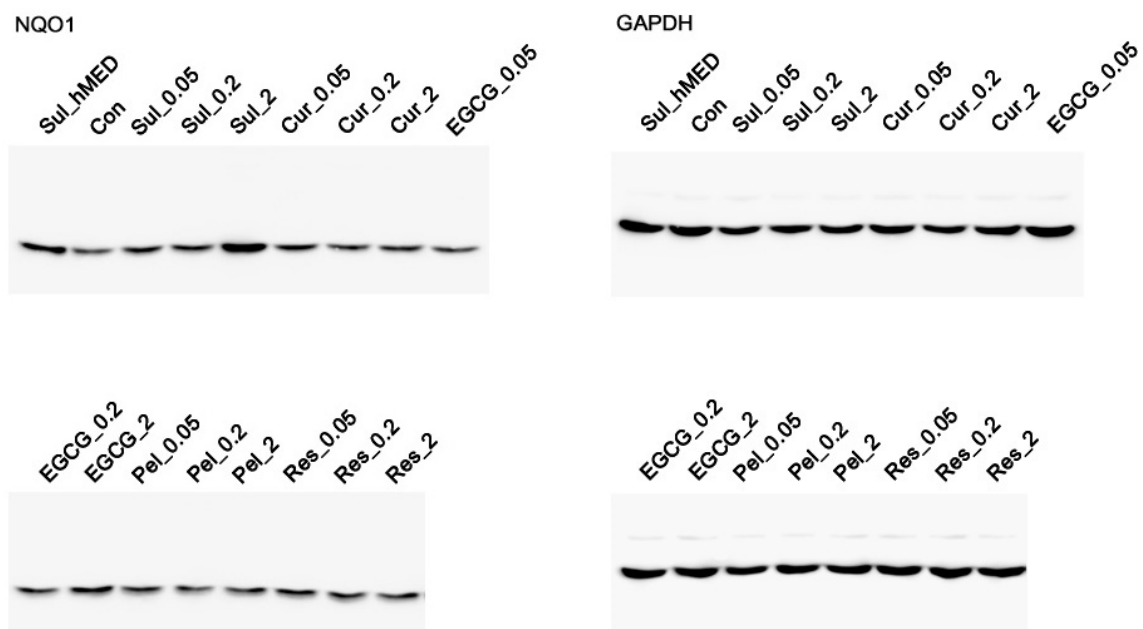

Figure 4

p-p38

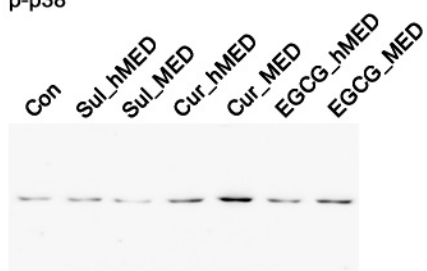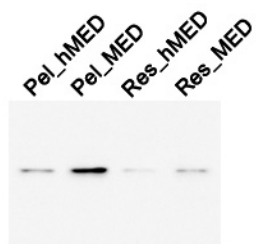

p38

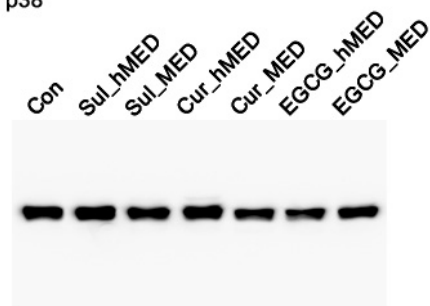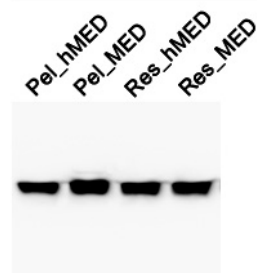

p-ERK

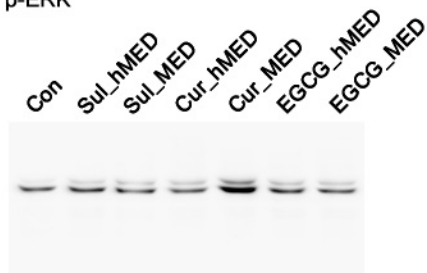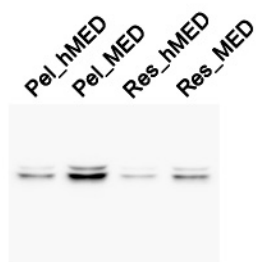

ERK

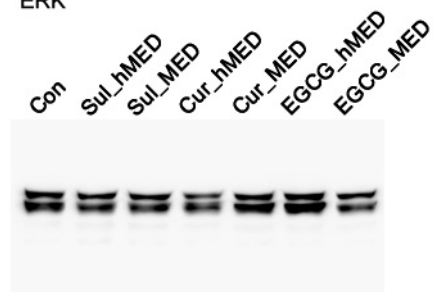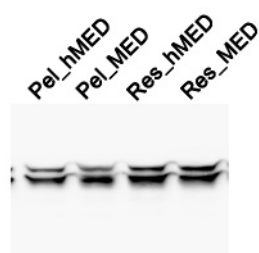

p-JNK

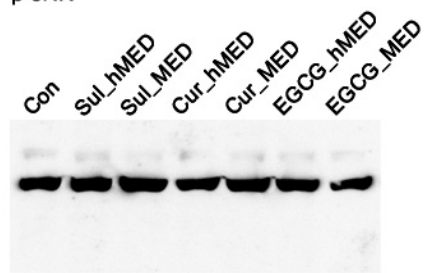

JNK

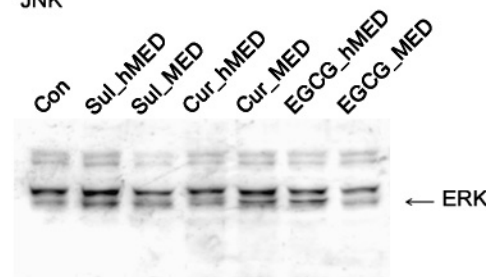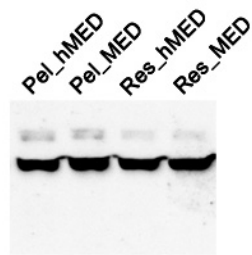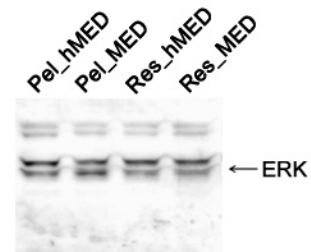

GAPDH

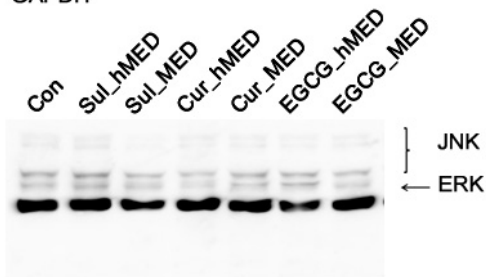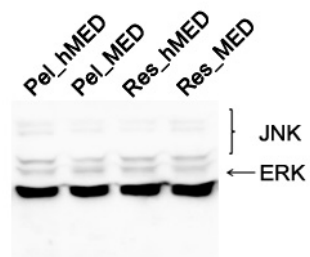

Figure 6

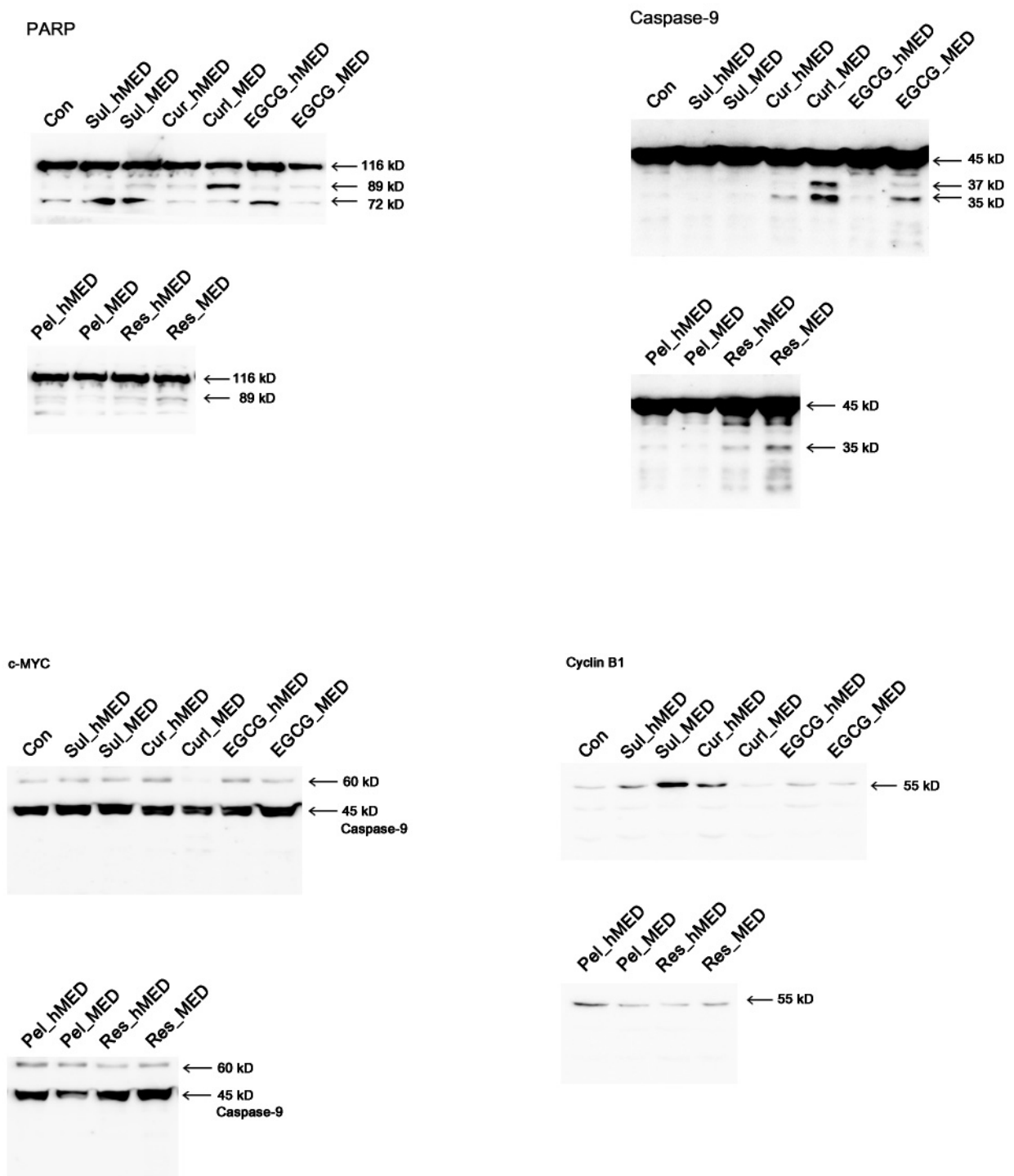

Cyclin D1

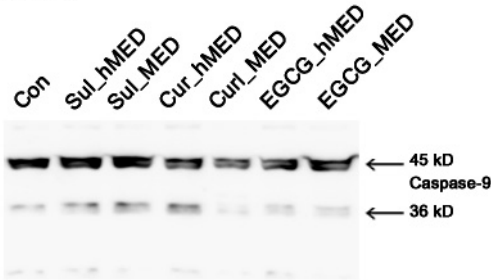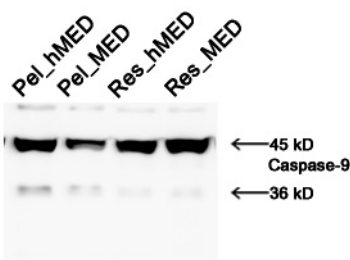

Cyclin E1

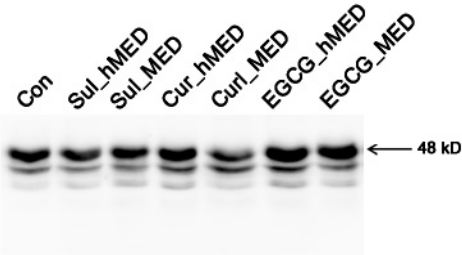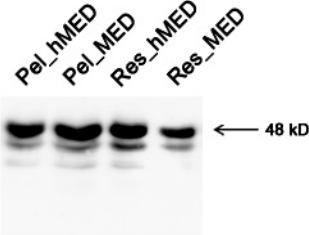

GAPDH

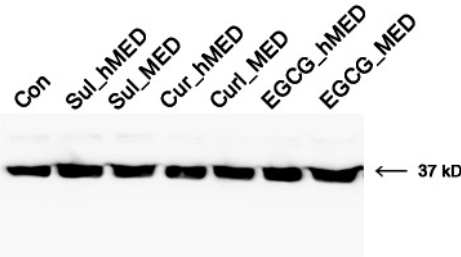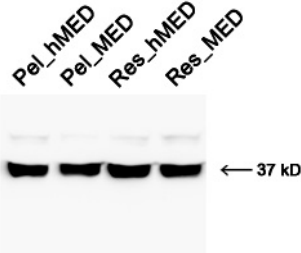

Supplement: Supplementary Figure 2 _spl_Full length blots for Figure 346_spl_ [file mmc1.pdf]
